# Genetic Diversity of Hepatic/Non-Hepatic Cystic Echinococcosis in Baqiyatallah Hospital, Tehran, Iran

**Published:** 2020

**Authors:** Hamidreza NEYSI, Tahereh MOHAMMADZADEH, Seyed Mahmoud SADJJADI, Jamal AKHAVANMOGHADDAM, Alireza SHAMSAEI

**Affiliations:** 1. Department of Parasitology and Mycology, School of Medicine, Baqiyatallah University of Medical Sciences, Tehran, Iran; 2. Health Research Center, Life Style Institute, Baqiyatallah University of Medical Sciences, Tehran, Iran; 3. Department of Parasitology and Mycology, School of Medicine, Shiraz University of Medical Sciences, Shiraz, Iran; 4. Department of Surgery, School of Medicine, Baqiyatallah University of Medical Sciences, Tehran, Iran; 5. The Laboratory of Pathology, Baqiyatallah Hospital, Tehran, Iran

**Keywords:** Genetic characterization, *Echinococcus granulosus*, Paraffin-embedded tissue, Iran

## Abstract

**Background:**

Cystic echinococcosis (CE) is a worldwide zoonotic helminthic disease caused by the larval stage of *Echinococcus granulosus*. The infection is particularly important in terms of economic and medico-veterinary aspects in endemic areas including Iran. Considering the possibility of organ-tropism in *E. granulosus* strains, the present study was aimed to identify the genotypes of *E. granulosus* in different organs involved in patients, undergone surgery in Baqiyatallah Hospital, Tehran, Iran from 2005–2015.

**Methods:**

Overall, 29 formalin-fixed paraffin-embedded tissues (FFPT) from patients with histologically confirmed CE including liver (N: 14) lungs (N: 6) abdomen (N: 2), pancreas (N: 2) and each of spleen, gallbladder and, muscles (N: 1) plus unknown organs (N: 2) were used and genetically characterized using polymerase chain reaction, followed by partial sequencing of mitochondrial cytochrome c oxidase gene subunit 1(cox1) and analyzed.

**Results:**

Nineteen out of 29 isolates including liver (N: 6) lungs (N: 4) abdomen (N: 2), pancreas (N: 2) and each of spleen, gallbladder, and muscle (N: 1), unknown organs (N: 2) obtained from paraffin-embedded blocks of human CE created an acceptable sequence in two directions. All 19 isolates regardless of the organ involved were recognized as *E. granulosus* sensu stricto (G1).

**Conclusion:**

The sequence alignments of the isolates displayed two profiles. All sequenced samples showed *E. granulosus* sensu stricto (G1) with no organ-related genotype.

## Introduction

Cystic echinococcosis (CE), is an important zoonotic parasitic disease with global distribution including Iran (1, 2). The infection is particularly important in terms of economic, medical and veterinary aspects in endemic areas (3). Human is usually infected following ingestion of parasite eggs via contaminated foods. The eggs develop to cysts in any organ especially in the liver and lungs (4). Diagnosis of hydatid disease is initially based on clinical signs followed by the imaging of suspected organs combined with serological tests (5–7). New techniques such as proteomics are also used (8). Diagnosis of the disease in human is difficult due to the absence of specific clinical signs; hence imaging methods may not be able to differentiate between hydatid cysts, tumors, and other lesions (9). Medical and surgical treatments are undertaken for the disease but surgery has been the most important and effective method for treating hydatid disease. However, the rupture of the cyst during surgery has been one of the major problems leading to the formation of secondary hydatid cysts in the abdominal cavity. Therefore, surgeons usually utilize several synthetic or natural protoscolicidal agents in treatment attempts to prevent this important complication (10–13).

Iran is an important region in the Middle East where various species of animals become infected by *E. granulosus* (2). Moreover, human CE is also documented in many studies in different parts of Iran (2, 6, 14, 15).

During recent years, the molecular characterization of several organisms such as different parasites has been developed in many areas. In this context, genotype characterization of *E. granulosus* could help different aspects of the echinococcosis including epidemiology, diagnosis, treatments and control strategies of this important disease (4, 15).

Five genotypes of *E. granulosus* including *E. granulosus* sensu stricto (G1–G3 complex), *E. ortleppi* (G5), and *E. canadensis* (G6) have been reported from animal and/or human in Iran (4, 15–21).

Considering the possibility of organ-tropism in *E. granulosus* strains, the present study was aimed to identify the genotypes of *E. granulosus* in different organs of patients’ undergone surgery in Baqiyatallah Hospital, Tehran, Iran.

## Materials and Methods

Overall, 29 out of 104 formalin-fixed paraffin-embedded tissues (FFPT) including liver (N: 14) lungs (N: 6) abdomen (N: 2), pancreas (N: 2) and each from spleen, gallbladder and muscles (N: 1) and also unknown organ (N: 2) from patients (19 males and 10 females) with histologically confirmed CE obtained from Baqiyatallah Hospital, Tehran, Iran from 2005–2015.

After xylene de-paraffinization and methanol rehydration (100%, 90%, 80%, 70 % and 60%) of the tissue sections from all samples, DNA was extracted using Tissue DNA Extraction Micro Kit (Favorgen, Taiwan) according to the manufacturer’s instructions. The quality of DNA was checked by NanoDrop (Biotek, USA, version: Epoch 2) and DNA was subsequently used as a template for polymerase chain reaction (PCR) using amplification of a partial mitochondrial DNA fragment of cox1. JB3 (TTTTTTGGGCATCCTGAGGTTTAT) and JB4.5 (TAAAGAAAGAACATAATGAAAATG) sequences were utilized as forward and reverse primers respectively (22). PCR was carried out in the final volume of 50 μL, including 4 μL (50–100 ng) of genomic DNA, 2 μL (25 p. mol) of each primers and 25μL of master mix including Taq DNA polymerase, Mgcl2, dNTP, PCR buffer and loading dye (Cinaclon, Iran) and 17 μL of DDW under the following conditions: 5 min at 94 °C as an initial denaturation step, followed by 35 cycles of 30 sec at 94 °C, 45 sec at 50 °C, 35 sec at 72 °C and a final extension step of 10 min at 72 °C. Negative (no added DNA) and positive controls were included in each PCR cycle. The amplification products were analyzed by electrophoresis in ethidium bromide-stained 1% agarose gel prepared in TAE buffered medium (65 mM Tris-HCl, 22.5 mM boric acid, 1.25 mM EDTA, pH 8.3) and subsequently visualized using an UV trans-illuminator (UVitec, Cambridge, UK).

A panel of 29 PCR amplicons for the cox1gene was purified using FavorPrep ^TM^ GEL/PCR Purification Kit (Favorgen, Taiwan) and subjected to sequencing in two directions, using the same PCR primer set (First BASE Laboratories Sdn Bhd-604944X, Malaysia).

The sequences of the cox1 gene were deposited in the GenBank database. Blast software was applied in order to preliminary identification and comparison of our sequences with other deposited ones in GenBank (http://www.ncbi.nlm.nih.gov). A Phylogenetic tree was drawn using our sequences and a few cases obtained from GenBank (Table 1). Alignment was carried out using ClustalW and the aligned sequences manually refined in BioEdit software (ver. 5.0.6) (23); maximum likelihood (ML) was inferred by MEGA 5 software for phylogenetic tree construction (24).

**Table 1:** The genotype of *Echinococcus granulosus* isolates identified by partial mitochondrial cox1 sequence in Baqiyatallah hospital (Tehran, Iran) and relevant information pertaining to the origins of sequences used for subsequent phylogenetic analyses (Fig. 2)

***Code (No)***	***Identification number of isolates***	***Geographic origin Country (City or County)***	***Organ localization***	***Accession Number***	***Strain (Genotype***	***References***
1	6451	Iran (Tehran)	Liver	MH244469	G1	Present study
2	13910	Iran (Melard)	Unknown	MH244470	G1	Present study
3	14207	Iran (Tehran)	Liver	MH244471	G1	Present study
4	3455	Iraq (not reported)	Liver	MH244472	G1	Present study
5	4119	Iran (Rasht)	Lung	MH244473	G1	Present study
6	6493	Iran (Kermanshah)	Lung	MH244474	G1	Present study
7	3865	Iran (Karaj)	Lung	MH244475	G1	Present study
8	1611	Iran (not reported)	Pancreas	MH244476	G1	Present study
9	2875	Iran (not reported)	Pancreas	MH244477	G1	Present study
10	510	Iran (not reported)	Abdomen	MH244478	G1	Present study
11	5262	Iran (not reported)	Unknown	MH244479	G1	Present study
12	560	Iran (not reported)	Muscle	MH244480	G1	Present study
13	360	Iran (Golestan-Bandar-e-Gaz)	Liver	MH244481	G1	Present study
14	644	Iran (Khomein)	Lung	MH244482	G1	Present study
15	7840	Iran (Pakdasht)	Liver	MH244483	G1	Present study
16	6355	Iran (Tehran)	Abdomen	MH244484	G1	Present study
17	2703	Iran (Baghmalek)	Liver	MH244485	G1	Present study
18	603	Iran (Tehran)	Gallbladder	MH244486	G1	Present study
19	1583	Iran (Tehran)	Spleen	MH244487	G1	Present study
20	KU37	Iraq (Kurdistan)	Not reported	MF004309*	G1	(34)
21	EG44	Peru	Not reported	AB688621*	G1	(10)
22	Not reported	Australia (Tasmania)	Not reported	M84662*	G2	(22)
23	Not reported	India	Not reported	M84663*	G3	(22)
24	KH10	Iran (Birjand)	Liver	KP751430*	G6	(21)
25	(Out group)	China: Inner Mongolia	Not reported	AB461420*	*E.m*	(35)

* GenBank samples for comparison

Approval of the study protocol was received from the Ethical Committee of Baqiyatallah University of Medical Sciences.

## Results

**Fig. 1:**
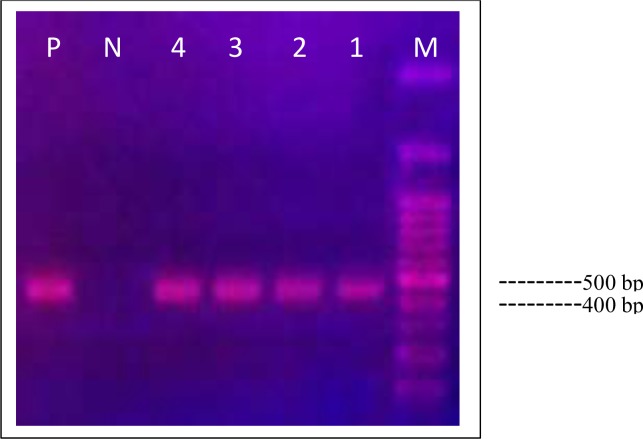
Gel electrophoresis of cox1–PCR products. Lanes 1–4: *E. granulosus* isolates; Lane N: negative control; Lane P: positive control; Lane M, 100 bp DNA ladder

Twenty-nine fragments of human CE isolates with around 450 bp long were successfully amplified within cox1 gene (Fig. 1).

Liver and lungs were the most organs involved with 14 (48.27%) and 6 (20.68%) cases respectively. Other organs have been shown to be 9(31.04%) including two in abdomen (6.9%), two in pancreas (6.9%), one (3.45%) in gallbladder, one (3.45%) in muscle and one(3.45%) in heart. Furthermore, the organ of the two (6.9%) isolates have not been reported.

**Fig. 2:**
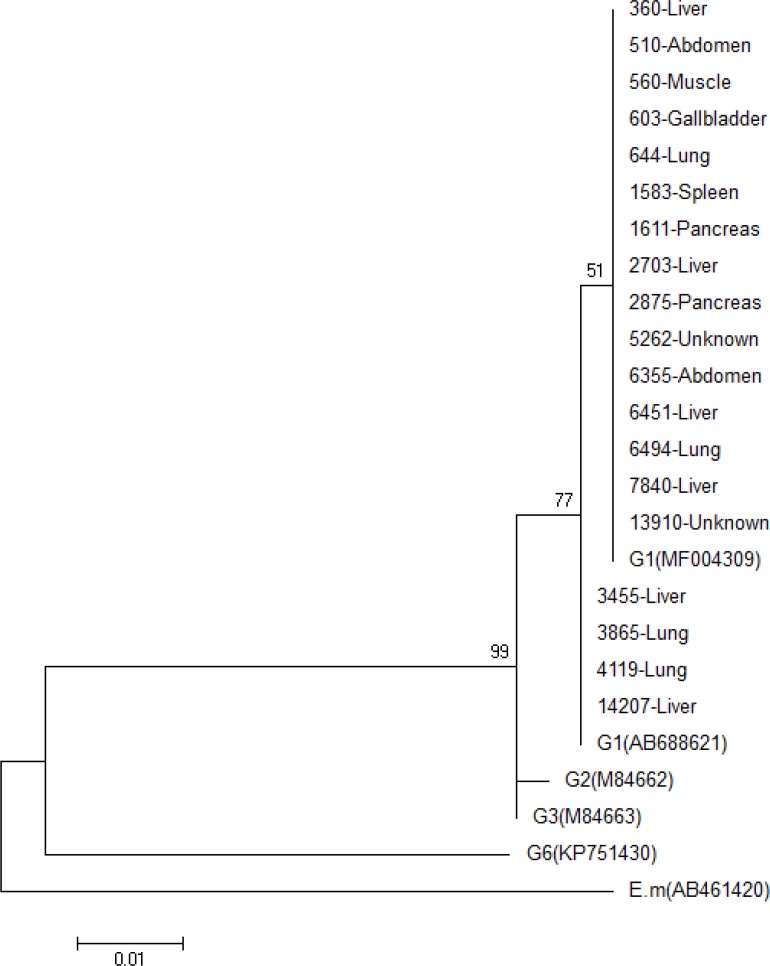
Genetic relationships of nineteen *Echinococcus granulosus* isolates from the Baqiyatallah hospital and selected GenBank sequence samples based on phylogenetic analysis of partial cox1 sequence data (Table 1). AB461420: *E. multilocularis* isolate from GenBank as the out-group

Nineteen out of 29 isolates including liver (N: 6) lungs (N: 4) abdomen (N: 2), pancreas (N: 2) and each of spleen, gallbladder and muscles (N: 1), and unknown organ (N: 2) obtained from paraffin embedded blocks of human CE resulted reasonable sequences in both directions. All 19 isolates regardless of their organs involved were recognized as *E. granulosus* sensu stricto (G1) (Table 1). The sequence alignments of the isolates displayed two profiles. Sequence profiles were deposited in GenBank with accession numbers MH244469–87. A consensus tree was made and the polymorphism points were shown (Fig. 2).

## Discussion

Despite many efforts in control and prevention of CE, the disease is one of the most important zoonotic tissue helminthic infections in the world especially in Mediterranean regions (25).

Different aspects of the disease including the molecular perspective of human and animal CE is useful for control programs. Recently many researchers have utilized molecular tools to access the information about parasite genotypic diversity (16–21, 26). Several genes including both coding and non-coding regions have used for intra-species differentiation; however, mitochondrial markers such as cox1 have shown great power in genetic discrimination (25–28).

The G6 genotype, which has already been reported as a main causative agent of brain CE in Iran (16–17); is widespread in Birjand, Eastern Iran (21).

Application of formalin-fixed, paraffin-embedded tissue samples have been used for discrimination of genotypes in different localities to show the geographical distribution of the genotypes (29).

In this context, in the present study 29 human CE samples obtained from various organs were successfully amplified within cox1 gene of which only 19 isolates including liver (31.58%) lungs (21.05%) abdomen (10.52%), pancreas (10.52%) and each of spleen, gallbladder and muscle (5.27%) plus unknown organ (10.52%) were shown an acceptable bidirectional sequences. All isolates regardless of the organ involved were recognized as *E. granulosus* senso strcto (G1) which supports previous reports as G1 as the more prevalent strain in Iran (15, 18–19, 29,30).

A study on formalin-fixed, paraffin-embedded tissue samples from Alborz, Tehran, and Kerman provinces on 125 samples reported 56%, 40%, 3.2% and 0.8% of isolates as G1, G6, G3, and G2 genotypes, respectively. Despite the high number of samples, there is no report of the organ involved in that investigation (29).

A study on 334 liver and lungs CE, originated from sheep, cattle, goats and human by sequencing of ITS1 genes in Khuzestan province showed only G1 strain in all tested samples (30). However, another study on human brain CE showed them as G6 while other samples from the human liver were reported as G1 strain which showed organ tropism in *E. granulosus* (16).

Molecular study on 30 samples from hospitalized patients in Isfahan showed them as G1 which is similar to our results (31). A study on the paraffin embedded of CE in Mazandaran Province showed the human isolates as *E. granulosus* sensu stricto (32).

Overall, 47 human CE from two hospitals in Tehran were amplified based on cox1 and nad1 genes. Sixteen (53.3%), 13 (43.3%), and 1 (3.3%) samples were related to three organs including lung, liver and spleen, respectively. Twenty-six and 3 of the 29 isolates were related to G1, and G3 strains, respectively. Only one G6 isolate was obtained from the lungs (33).

The lack of diversity in the isolates of our study could be because G1 is a common strain worldwide consequently, Iran and the number of samples although from different geographical origin (Tehran, Golestan, Kermanshah, Central provinces and even Iraq) is parallel to the most prevalent strain in those areas.

## Conclusion

Genetic diversity of hepatic and non-hepatic cystic echinococcosis in Baqiyatallah Hospital, Tehran, during ten years showed that *E. granulosus* sensu stricto is predominant. This means that more attention should be directed to the diagnosis and treatment protocols to this important genotype in this geographic region.
